# Studies on Biochemical Changes in Subacute Thiodicarb Toxicity in Rats

**DOI:** 10.4103/0971-6580.68347

**Published:** 2010

**Authors:** Satpal, S. K. Jain, J. S. Punia

**Affiliations:** Department of Pharmacology and Toxicology, CCS Haryana Agricultural University, Hisar - 125 004, Haryana, India

**Keywords:** Biochemical changes, rats, subacute toxicity, thiodicarb

## Abstract

Effect of thiodicarb was investigated on various biochemical parameters and blood enzymes in adult male Wistar rats following its intraperitoneal administration at rates of 2.9 and 5.8 mg/kg daily for 28 days. Rats did not exhibit any marked changes in their gross behavioral signs and symptoms. Thiodicarb caused hyperglycemia in rats; however, increase in plasma glucose level was nonsignificant. There was no effect on total plasma protein indicating no severe damage to vital organs and no interference with protein metabolism in rats. Thiodicarb did not cause significant change in blood urea and creatinine levels, thus indicating to have no toxic effect on kidneys in rats. It did not affect aspartate aminotransferase (AST) level except a significant increase in AST level only on 7th day of treatment. There was an increase in the levels of alanine aminotransferase (ALT), but this trend reversed on 14th and 28th day. Thiodicarb did not alter significantly the levels of alkaline phosphatase in rats. It caused inhibition of plasma and brain acetylcholinesterase (AChE) in rats throughout the entire period of 28 days of treatment, which was dose-dependent. The findings of this investigation indicated that thiodicarb did not effect or alter much the various biochemical profiles except inhibiting AChE following i.p. administration up to 28 days in adult male rats.

## INTRODUCTION

Pesticides are extensively and indiscriminately used in the modern agricultural practices resulting in widespread distribution in the environment and posing serious health hazards to animals and human beings. Besides inhalation from polluted environment, animals are also exposed to pesticides through utilization of treated feeds and fodders. Thiodicarb (dimethyl *N*, *N’* -thiobis (methyl imino) carbonyloxy bisethanimido thioate) is a new carbamate compound having broad spectrum of activity being extensively used for the crop protection. It is a class II category compound (moderately toxic) as set forth by the United States Enviromental Protection Agency (USEPA) and World Health Organization (WHO). Various carbamate compounds have been reported to cause biochemical changes in different species of animals.[1–5] Little information on the effect of thiodicarb on biochemical profiles is available in dogs and rats.[6–8] However, no detailed report is available about the effects of thiodicarb on various biochemical parameters and blood enzymes in animals. Therefore, the present investigation was undertaken to study the effects of thiodicarb on various biochemical parameters following daily intraperitoneal (i.p.) administration for 28 days in adult male Wistar rats.

## MATERIALS AND METHODS

Fifty-four adult male Wistar rats weighing about 125 g each were procured from Disease Free Small Animal House of the University and kept in the departmental small animal house for 4 days prior to experimentation for acclimatization. The animals were given standard feed and water *ad libitum*. Animals were divided into nine groups each consisting of six rats. Three groups of six rats each served as controls receiving normal saline (*n* = 18) i.p. daily and remaining six groups of six rats each received thiodicarb (Larvin 75 WP, Rhone Poulenc India Ltd., Mumbai) at the dose rates of 1/10th (*n* = 18) and 1/5th (*n* =18) of LD _50_, i.e., 2.9 and 5.8 mg/kg, respectively, i.p. daily till sacrificed on the 7th, 14th, and 28th day of the treatment (six on each day from the control and two dose groups). Rats were anesthetized with ether and blood samples were taken directly from the heart in a heparinized syringe after opening the chest.

Different biochemical parameters viz. blood glucose, total protein, blood urea, plasma creatinine, and activities of various blood enzymes namely aspartate aminotransferase (AST), alanine aminotransferase (ALT), and alkaline phosphatase (ALP) were determined by autoanalyzer (Hitachi Model 905), using the reagent kits of Transasia. Activity of acetylcholinesterase (AChE) enzyme in plasma and brain was determined by microassay method[9] and expressed as enzyme activity/min/ml plasma or activity/min/g tissue. The data were analyzed using the Duncan’s Multiple Range Test.[10]

## RESULTS AND DISCUSSION

None of the adult male rats exhibited any marked change in the gross behavioral signs and symptoms receiving thiodicarb at the dose rates of 2.9 and 5.8 mg/kg i.p. daily for 28 days. The food and water consumption by the control and treated rats remained almost completely normal during the entire period of study.

The effects of thiodicarb on blood glucose, total protein, blood urea, and creatinine in rats are presented in Table 1. Thiodicarb caused hyperglycemia in rats. The maximum increase in plasma glucose level was observed on 28th day. However, increase in glucose level was nonsignificant (*P*<0.05). This might be due to the release of catecholamines after insecticide administration stimulating adenylate cyclase enzyme and thus increasing cyclic AMP levels, which stimulates phosphorylase enzyme, which further stimulates carbohydrate metabolism leading to hyperglycemia.[11] It may also act as chemical stressor increasing the secretion of glucocorticoids, thus stimulating gluconeogenesis.[12] Similar observations have been demonstrated in rabbits and dogs administered carbaryl[13] and rats given carbofuran.[1] In cholinesterase inhibitors poisoning, asphyxia occurs due to impaired respiration leading to increased discharge of sympathetic nervous system.[14] The asphyxia acts as primary stimulus for glycogenolysis and hyperglycemic effect.[15]

**Table 1:** Effect of thiodicarb (i.p. daily for 28 days) on plasma glucose, total protein, blood urea and creatinine in adult male Wistar rats

Parameter (mg/kg)	Dose	Days		
		7	14	28
Plasma	Control	126.0±11.7	116.7±11.2	99.2±11.7
glucose	2.9	122.6±19.6	124.0±6.4	110.0±15.1
(mg/100 ml)	5.8	139.2±13.6	127.3±8.4	130.7±21.2
Total	Control	6.97±0.25	6.23±0.05	7.00±0.06
Protein	2.9	7.18±0.48	6.44±0.91	6.85±0.12
(g/100 ml)	5.8	7.08±0.56	6.15±0.05	6.65±0.17
Blood urea	Control	18.83±3.27	25.83±2.04	25.01±3.21
(mg/100 ml)	2.9	16.80±1.96	24.40±1.60	18.20±2.83
	5.8	16.33±0.80	27.50±1.63	25.50±3.68
Creatinine	Control	0.87±0.05^A^	0.91±0.05	1.20±0.10
(mg/100 ml)	2.9	0.74±0.06^AB^	0.88±0.04	0.80±0.20
	5.8	0.70±0.03^B^	0.95±0.06	1.13±0.09

Values are mean ± SE of six animals; Means bearing different superscripts differ significantly (*P*<0.05)

There was no change in total plasma protein content in rats at any dose level of thiodicarb, indicating no severe damage to vital organs and also no interference with protein metabolism. Our findings are in agreement with similar observations in rats given carbofuran.[1] However, a decrease in total protein and globulin has been observed in dogs given thiodicarb for 6 months.[3] Thiodicarb did not cause significant change in the blood urea levels at any of the dose level. Only slight increase in blood urea level was observed on 14th and 28th day of treatment as compared to control, but it was within normal range. There was a decrease in plasma creatinine level on 7th and 28th day. Since, thiodicarb did not cause significant elevation in blood urea or creatinine levels, it indicated to have no toxic effect on kidneys. Similar observations have been reported in chicks fed on butocarboxim for 30 days.[4] Contrary to this, an increase in blood urea nitrogen levels has been reported in rats and chicks given benfuracarb.[5,16]

Thiodicarb did not affect much the plasma AST levels in rats, except a significant increase (192.8 IU/L) on 7th day of treatment as compared to control rats (134.3 IU/L). There was an increase in the levels of ALT on 7th day, but this trend reversed on 14th and 28th day [Table 2]. The activity of aminotransferases in blood is generally low, but it increases rapidly following trauma or necrosis of heart muscles, skeletal muscles, or hepatic tissues, as these enzymes diffuse across the damaged cell wall and then enter the circulation. Elevation of aminotransferases in blood is used as an indicator of tissue damage and altered plasma membrane permeability.[17] Our findings indicated that thiodicarb did not adversely affect or cause specific damage to vital organs, particularly liver and heart. This is further supported by our findings where no marked histopathological changes in liver and heart in rats given thiodicarb were seen.[18] However, thiodicarb resulted in an increase in AST and ALT levels in dogs given at a rate of 45 mg/kg/day for 6 months.[6] An increase in AST and ALT levels has been reported in carbaryl toxicity in rats.[1,2]

**Table 2:** Effect of thiodicarb (i.p. daily for 28 days) on the activities of blood enzymes in adult male Wistar rats

Parameter (mg/kg)	Dose	Days		
		7	14	28
AST	Control	134.3±4.4^B^	110.5±22.1	192.8±28.0
(IU/L)	2.9	192.8±19.5^A^	106.2±15.7	227.2±11.6
	5.8	133.8±17.9^A^	92.1±5.4	116.8±11.5
ALT	Control	28.3±3.1	57.0±4.7	64.2±6.6
(IU/L)	2.9	67.4±9.8	41.8±5.5	46.0±2.7
	5.8	47.1±7.9	46.3±3.0	49.8±4.3
ALP	Control	6.16±1.79	3.33±0.21^B^	3.50±0.22
(IU/L)	2.9	4.25±0.25	4.00±0.12^A^	3.92±0.07
	5.8	4.26±0.11	4.00±0.20^A^	3.70±0.16
Plasma	Control	1.34±0.02^A^	1.32±0.02^A^	1.36±0.03^A^
AChE	2.9	1.17±0.04^B^	1.10±0.05^B^	1.05±0.02^B^
	5.8	1.09±0.02^B^	0.95±0.02^C^	1.01±0.02^B^
Brain	Control	1.06±0.06^A^	1.13±0.03^A^	1.12±0.04^A^
AChE	2.9	0.92±0.03^AB^	0.89±0.03^B^	0.88±0.04^B^
	5.8	0.88±0.04^B^	0.83±0.04^B^	0.78±0.05^B^

Values are mean±SE of six animals; Means bearing different superscripts differ significantly (*P*<0.05)

Thiodicarb caused reduction in the plasma level of alkaline phosphatase (ALP) in rats on 7th and 28th day of treatment as compared to control [Table 2]. However, marked elevation in ALP levels has been reported in rats given benfuracarb.[5] Damage to liver, small intestine, bone, and kidney may increase the levels of ALP in blood.[19] Since, there was no significant effect in the ALP levels, the study indicated that thiodicarb did not cause appreciable damage to vital organs in the treated rats. This is further supported by our findings where no marked histopathological changes were observed in liver and kidney in rats given thiodicarb.[18]

Thiodicarb caused significant inhibition of plasma and brain acetylcholinesterase (AChE) activity in rats at both the dose levels during the entire period of experiment of 28 days [Table 2]. The reduction in plasma AChE activity was observed to be much more at high dose of thiodicarb. Similar findings have been reported in dogs and rats after oral and dermal application of thiodicarb.[7,8]

On the basis of these findings, it can be inferred that thiodicarb did not cause significant alterations in the activities of most of the biochemical profiles and blood enzymes except AChE inhibition on repeated intraperitoneal administration in rats.
